# MicroRNAs and Molecular Mechanisms of Neurodegeneration

**DOI:** 10.3390/genes4020244

**Published:** 2013-05-29

**Authors:** Ilaria Bicchi, Francesco Morena, Simona Montesano, Mario Polidoro, Sabata Martino

**Affiliations:** Department of Experimental Medicine and Biochemical Sciences, University of Perugia, Via del Giochetto, 06126 Perugia, Italy; E-Mails: ilaria.bicchi@virgilio.it (I.B.); effemorena@gmail.com (F.M.); simona.montesano82@gmail.com (S.M.); mapoli_2000@yahoo.com (M.P.)

**Keywords:** miRNAs, Alzheimer’s disease, Parkinson’s disease, Huntington’s disease, Amyotrophic Lateral Sclerosis, Lysosomal Storage Disorders

## Abstract

During the last few years microRNAs (miRNAs) have emerged as key mediators of post-transcriptional and epigenetic regulation of gene expression. MiRNAs targets, identified through gene expression profiling and studies in animal models, depict a scenario where miRNAs are fine-tuning metabolic pathways and genetic networks in both plants and animals. MiRNAs have shown to be differentially expressed in brain areas and alterations of miRNAs homeostasis have been recently correlated to pathological conditions of the nervous system, such as cancer and neurodegeneration. Here, we review and discuss the most recent insights into the involvement of miRNAs in the neurodegenerative mechanisms and their correlation with significant neurodegenerative disorders.

## Abbreviations

**Table:** 

Ago2 = Argonaute-2	ALS = Amyotrophic Lateral Sclerosis
APP = Amyloid precursor protein	BACE1 = beta-site APP-cleavage enzyme 1
BTBD3 = BTB (POZ) domain containing 3	COL2A1 = Collagen, type II, alpha 1
CoREST = REST Corepressor	COXIV = cytochrome c oxidase IV subunit
dAgo1 = Drosophila Argonaute-1	DAT = Dopamine transporter
DJ1 = Parkin-7	E2F1/DP = E2F transcription factor 1
EAAT2 = excitatory amino acid transporter 2	TDP-43 = TAR DNA-binding protein 43
GALC = galactosylceramide	GLT-1 = glutamate transporter
hAgo2 = human Argonaute-2	HDAC4 = Histone Deacetylase 4
HTT = Huntingtin	LSD = Lysosomal Disease
NEFL = Neurofilament light polypeptide	NfKB = nuclear factor kappa BNPC = Niemann Pick cells
PACT = protein activator of PKR	TBP = Tata Binding Protein
phospho-4E-BP1 = 4E binding protein1	Pitx3 = paired-like homeodomain transcription factor 3
PTBP2 = Polypyrimidine tract binding protein 2	RLC = regulatory light chain
SH-SY5Y = Human Neuroblastoma Cells Line	SIRT1 = sirtuin 1
SNP = single-nucleotide polymorphism	SPT = Serine palmitoyltransferase
TAp73 = Tumor protein p73	ERK1 = Extracellular signal-regulated kinase 1FUS/TLS = fused in sarcoma/traslocated in liposarcoma
PGC-1α = Peroxisome Proliferator—Activated Receptor Gamma Coactivator 1	TGFBI = transforming growth factor, beta 1TLR-7 = Toll-like receptor 7
TRBP = Tar RNA binding proteinTRIM2 = tripartite motif containing 2	

## 1. Biogenesis and Role of microRNAs

MicroRNAs (miRNAs) are a class of ~22 nucleotides non-coding RNA molecules representing a superior mechanism by which to regulate gene expression.

MiRNAs biosynthesis is conserved during evolution and consists of two steps that take place in the nucleus and the cytoplasm, respectively [1,2].

In the *nucleus*, miRNAs are largely transcribed by RNA polymerase II as primary-(pri-)miRNAs. At this stage, pri-miRNAs present several hairpin structures, each consisting of a stem and a terminal loop, and are subject to a 5'-capping, 3'-polyadenylation, editing, and splicing processing [3,4]. The processed pri-miRNAs are next “cropped” into smallest hairpin-structures precursor of ~70 nucleotides (called pre-miRNAs) by a nuclear microprocessor complex composed of Drosha, an RNase III enzyme (RNASEN), and DGCR8 (DiGeorge Critical Region 8) protein. The last is also called Pasha (Partner of Drosha) in *D. melanogaster* and *C. elegans* [5]. These proteins form a complex with several cofactors (e.g., DEAD box RNA helicases p68 [DDX5]; p72 [DDX17]; heterogeneous nuclear ribonucleo-proteins [hnRNPs]) important for the specificity of Drosha activity [6]. Due to RNase activity, Drosha cleaves the 5' and 3' arms of the pri-miRNA hairpin [7], while DGCR8 is necessary for the interaction with the pri-miRNA for the site-specific cleavage [8]. Thus, Drosha cleaves 11 base pairs away from the single-/double-stranded RNAs at the level of the hairpin stem base [8]. The cleavage occurs co-transcriptionally [7,8,9,10] and generates a product with 2 nucleotides with 3' overhang that is specifically recognized by Exportin-5, which transports the pre-miRNAs into the cytoplasm via a Ran-GTP-dependent mechanism [4,11].

Alternatively, miRNAs may be generated by splicing and debranching of short hairpin introns [12,13] called “MiRtrons”, or by processing of small nucleolar RNAs (snoRNAs), transfer RNAs (tRNAs), and endogenous short hairpin RNAs (shRNAs) using a microprocessor complex independent route [14,15,16,17,18,19].

In the *cytoplasm*, the pre-miRNA enters into the RISC Loading Complex consisting of Dicer (RNase), the double-stranded RNA-binding domain proteins TRBP, PACT and the core component Ago2 [6,20,21,22]. Dicer, TRBP, and PACT process pre-miRNAs to ~22 nucleotides long miRNAs duplex [20,21,22,23]. The two miRNA strands are then separated and the *guide* strand is associated with an Argonaute protein within the RISC, where it is directly involved in the silencing of target messages.

Thermodynamically the miRNAs duplex is asymmetric [24,25]. As a consequence, miRNA strand whose 5'-end is less stably base-paired will usually be chosen as the strand *guide*. In contrast, the miRNA strand of which the 5'-end is more stably base-paired (the *passenger* strand) will be excluded from the RISC Loading Complex and generally degraded [3,4,26].

### 1.1. Canonical Function of microRNAs

**Figure 1:**
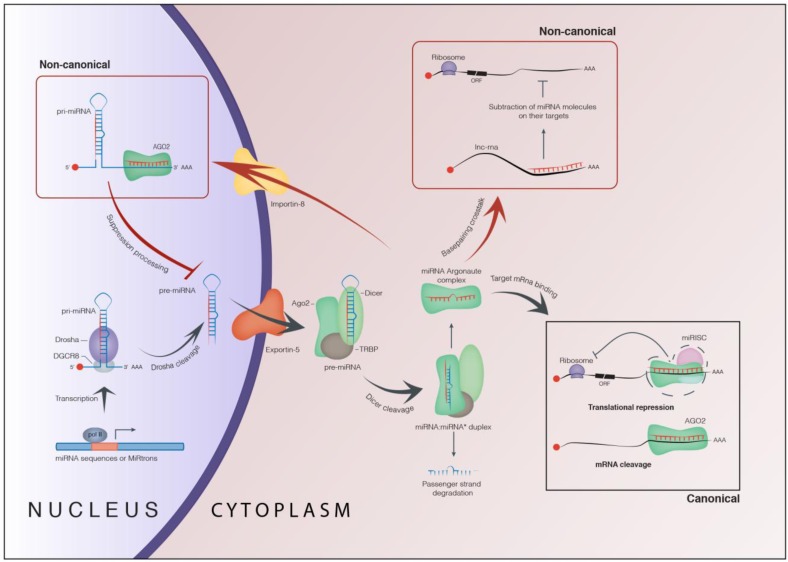
Biogenesis and function of microRNAs. Image shows the most relevant nuclear and cytoplasm steps of the biogenesis of miRNAs together with the canonical and non-canonical activity of miRNAs (see main text for details).

MiRNAs drive RISC to complementary sites within the target mRNAs in order to mediate their repression at the post-transcriptional level trough RNA-RNA base pairing, or translational repression, and/or mRNA deadenylation and decay (Figure 1) [1,27,28,29,30].

MiRNAs bind to their cognate target mRNAs in the site-specific sequences, called miRNA Recognition Element (MRE), through a mechanism based on the pairing of the “seed” sequence involving ~6–8 nucleotides at the 5'-end of the miRNAs [31].

### 1.2. Non-Canonical Function of microRNAs

Recent studies have shown that miRNAs are also re-imported, perhaps, via exportin-1 or importin-8, from the cytoplasm to the nucleus through a combination with Argonaute proteins. Here, miRNAs could regulate gene expression at the transcriptional level (Figure 1) [32,33,34]. 

Additionally, evidence has highlighted a new regulatory circuit in which miRNAs can crosstalk each other through a new smart “biological alphabet” represented by the “MRE” sequences that “act as the *letters* whose different combinations may form an entire universe of *words*” (from Salmela *et al.* 2011 [35]). In detail, Pandolfi’s hypothesis has proposed that mRNAs, miRNAs, transcribed pseudogenes, and long noncoding RNAs (lncRNA, a class of non-protein coding transcripts, usually 200 to 1,000 of nucleotides in length) using MRE sequences “talk” to each other and suggested that this “competing endogenous RNA” (ceRNA) activity forms a large-scale regulatory network across the transcriptome [35], and acts as player in the human genome for regulating the distribution of miRNAs molecules toward specific targets. This mechanism is straightforward for physiological and pathological processes [35,36,37,38,39,40,41,42].

## 2. MicroRNAs and Neurodegeneration

Neurodegenerative diseases are a group of late onset progressive disorders of the nervous system, characterized by a complex pathogenesis that generally involves multiple basic cellular pathways alterations [43,44,45,46,47,48,49,50,51,52,53]. Thus, understanding the wide spectrum of cell mechanisms could be relevant for the development of more effective therapies for these disorders.

Emerging evidence addresses a key role of non-coding RNAs in neurogenesis and neurodegeneration [45,46,47,48]. This review discusses the current advancements on miRNAs and neurodegenerative processes. Here we summarized the most recent insights in the issues collected from some selected neurodegenerative diseases: Alzheimer’s disease (AD) [49], Parkinson’s disease (PD) [50], Amyotrophic Lateral Sclerosis (ALS) [51], and polyglutamine (polyQ) disorders such as Huntington’s disease (HD) [52] and Lysosomal Storage Disorders (LSD) [53].

Table 1 reports a landscape of miRNAs that are considered implicated at different levels in AD, PD, HD, ALS, and LSD pathogenesis. Overall, these findings highlight the critical impact of select miRNAs on regulating the expression of chief proteins in neurodegeneration (both pathogenesis and progression).

**Table 1:** Reports a landscape of miRNAs involved in the pathogenesis of Alzheimer’s disease (AD), Parkinson’s disease (PD), Huntington’s disease (HD), Amyotrophic Lateral Sclerosis (ALS), and Lysosomal Storage Disorders (LSDs) not included in the main text.

microRNA	Neurodegenerative Disease	Molecular Target	Effects	Reference
miR-15	AD	ERK1 and Tau	ERK1 and Tau regulation	[54]
miR-16	AD	APP	Overexpression reduced APP level	[55]
		ERK1 and Tau	ERK1 and Tau regulation	[54]
miR-106a	AD	APP	APP repression	[56]
miR-106b	AD	APP	Aberrantly expressed in APPswe/PSE9 mice	[57]
miR-107	AD	BACE1	Downregulated.	[58]
			Repression of Cofilin translation, a component of rod-like actin structures in the AD brain.	
miR-124	AD	BACE1	Suppressed induces over expression of BACE1	[59]
miR-132	AD	PTBP2	Neuronal splicing regulator of Tau Exon 10	[54]
miR-137	AD	SPT	SPT and in turn Aβ levels up-regulate	[60]
miR-153	AD	APP	Downregulated in modest AD pathology	[56]
miR-195	AD	BACE1	Overexpressed decreased BACE1 protein level	[61]
miR-497	AD	ERK1 and Tau	ERK1 and Tau regulation	[54]
miR-520c	AD	APP	APP repression	[56]
Let-7b	AD	TLR-7	Induce Toll-like receptor 7 activation	[62]
miR-7	PD	α-synuclein mRNA	It can represses α-synuclein protein levels collaborating with miR-153	[63]
miR-133b	PD	Pitx3	Downregulated in PD brain tissue	[64]
miR-34b/c	PD	SH-SY5Y dopaminergic neuron	Downregulated	[65]
miR-let7	PD	LRRK2	Regulation of Drosophila e2f1 protein synthesis: repressed expression	[66]
miR-184	PD	LRRK2	Regulation of dp messenger RNAs synthesis: repressed expression	[66]
miR-433	PD	SNP rs12720208 in the 3' UTR	Increased FGF20 expression and upregulation of alpha-synuclein	[67]
miR-9/miR-9*	HD	REST/COREST	Downregulated. Double negative feedback loop between the REST silencing complex and the miRNAs it regulates	[68]
				[69]
miR-29c	HD	REST	Downregulated	[70]
miR-34b	HD	p53	Mysregulated causing by mHTT accumulation. Overexpressed in plasma of HD patients	[71]
miR-124	HD	REST	Downregulated leads to an increases of their target level	[68]
miR-125b	HD	HTT	Downregulated	[72]
miR-222	HD	REST	Downregulated.	[70]
miR-132	HD	REST	Downregulated. Neurite sprouting	[68]
miR-135	HD	REST	Downregulated	[68]
miR-137	HD	REST	Aberrantly repressed directly mediated by REST	[73]
miR-146a	HD	TBP	Regulation of TBP by miR-146a may contribute to HD pathogenesis. Generally downregulated	[72]
miR-150	HD	HTT	Downregulated	[72]
miR-153	HD	REST	Downregulated	[73]
miR-200a	HD	Genes regulating synaptic function, neurodevelopment, and neuronal survival	Upregulated. Perturbed expression in HD patients.	[74]
miR-200c				[74]
miR-9	ALS	NEFL	Downregulated	[75]
miR-23a	ALS	PGC-1	Upregulated. It can reduce PGC-1α signalling, cytochome-b and COXIV protein levels	[76]
miR-29b	ALS	p53	Upregulated	[76]
miR-124	ALS	EAAT2/GLT1	Indirect miR-124a-mediated regulation of GLT1 expression from neurons to astrocytes	[77]
miR-206	ALS	HDAC4	Upregulated in ALS end stage model to regenerate damaged neuromuscular synapses by HDAC4 reinnervation via	[78]
miR-455	ALS	COL2A1	Upregulated in skeletal muscles of ALS patients	[76]
Let-7b	ALS	TDP-43	Downregulated	[79]
miR-663	ALS	TDP-43	Upregulated	[79]
miR-126	LSD	GALC	Expressed in HSCs but not in differentiated cells	[80]
miR-130				[80]
miR-196a	NPC	Lipid biosynthesis associated genes	Upregulated	[81]
miR-196b	NPC	Lipid biosynthesis associated genes	Upregulated	[81]
miR-296	NPC	Lipid biosynthesis associated genes	Upregulated	[81]
miR-98	NPC	Lipid biosynthesis associated genes	Downregulated.Lipid biosynthesis associated	[81]
miR-143	NPC	Lipid biosynthesis associated genes	Downregulated.	[81]
			Lipid biosynthesis associated	

### 2.1. MicroRNAs and Alzheimer’s Disease

The pathological hallmarks of AD are the deposition of intracellular neurofibrillary tangles containing Tau protein and the accumulation of extracellular plaques containing β-Amyloid (Aβ) peptides, beginning in the hippocampus, and spreading progressively throughout the brain [82,83,84]. The basic mechanisms generating Aβ are largely studied and now include microRNAs. This emerges by growing evidence suggesting that alterations in the miRNA network could contribute to risks for AD (Table 1). Here we discuss some recent significant examples.

**Figure 2:**
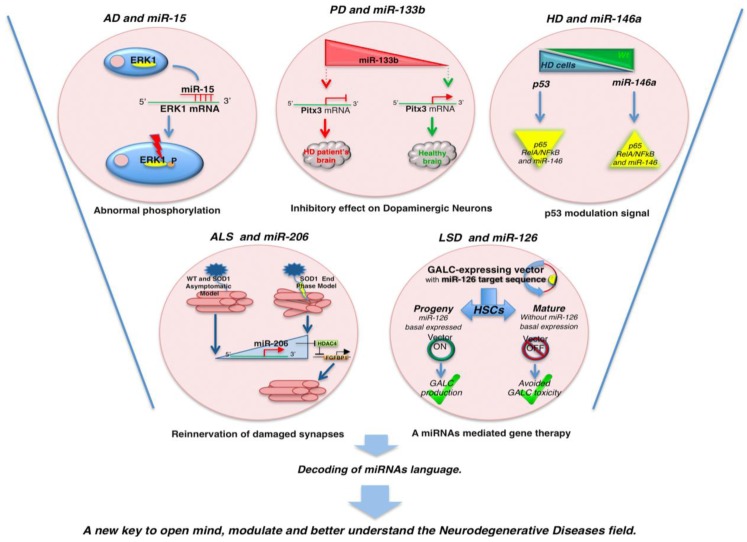
MicroRNAs and neurodegenerative mechanisms. Image shows some selected example of miRNAs involved in the pathogenesis of AD, PD, HD, ALS, and LSDs. Details of each mechanism are described in the text.

The TargetScan program revealed that several members of the miR-16 family (miR-16, miR-15, miR-195, miR-497) could modulate endogenous ERK1 and Tau phosphorylation in neuronal cells *in vitro*. Of note, miR-15 directly targets ERK1. Given that ERK1 is a Tau kinase, alteration of miR-15 is considered as a potential cause of the abnormal Tau phosphorylation observed in AD (Figure 2) [54]. Indeed, the same research group demonstrated that miR-132 is also involved in the regulation of the splicing of the endogenous Tau exon 10 by targeting PTBP2 in the neurons [54].

miR-153 levels were significantly reduced in the brains of a subset of human AD specimens with mild AD pathology. The same group of patients also exhibited increased levels of β-Amyloid precursor protein (APP), compared to healthy controls. Using bioinformatics algorithms, Long *et al.* revealed that miR-153 had a target site within the 3'-untranslated region (3'-UTR) APP mRNA. These computational results were confirmed by *in vitro* and *in vivo* experiments. Interestingly, miR-153 also caused the reduction of the expression of the APLP2 (APP paralog), indicating that miR-153 could be a player for biological pathways involving both proteins [85,86].

**Figure 3:**
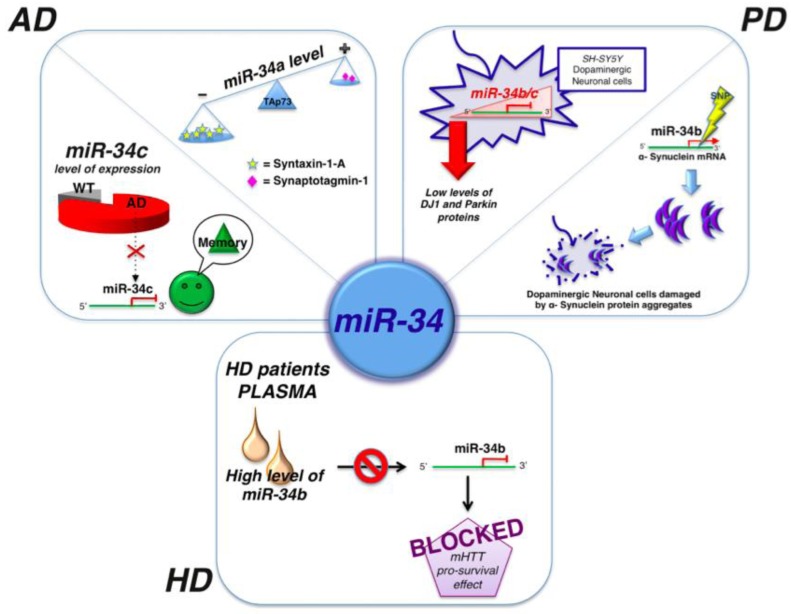
miR-34 and neurodegeneration. Image shows the correlation of miR-34a, miR-34b, and miR-34c and AD, PD, and HD. Details of each mechanism are described in the text.

Other studies indicated miR-34c as a potential marker for the cognitive disturbances associated with AD. miR-34c levels were elevated in the hippocampus of AD patients and corresponding mouse models (Figure 3). In fact, inhibition of miR-34c activity correlated with restoration of molecular mechanisms involved in the observed improvement of memory. Thus, it was suggested that miR-34c could be a negative control of memory consolidation [87]. Similar studies demonstrated the increase of the expression of miR-34a in the brain cortex of AD mouse models [88,89] and a specific interplay among miR-34a, TAp73, and synaptotagmin-1. TAp73 is a p53-family member and drives the expression of miR-34a, but not miR-34b and miR-34c, by acting on specific binding sites to the miR-34a promoter [89]. In brains of AD patients, the miR-34a, TAp73, and synaptotagmin-1 circuit is conserved although the aberrant expression of p73 (Figure 3) [89].

Several microRNAs target APP. In this context, miR-29a and miR-29b that target the APP and BACE1 mRNAs in their 3'-UTRs, are down regulated in a subset of AD patients. As a consequence, BACE1 protein expression is increased and the amyloidogenic peptide generated. This phenomenon was particularly evident in neuronal and glial cells indicating that the derepression of BACE1 expression that can occur in neurons or astrocytes [90]. Indeed, the BACE1 3'-UTR is also the target of miR-107, miR-124, and miR-195 (Table 1).

Interestingly, miR-107 controls the expression of other proteins relevant to AD pathology, such as cofilin [58], an actin-binding protein that accumulates in cytoplasmic inclusions known as Hirano bodies [91] (Hirano, 1994). This indicated that a single miRNA deregulation could activate several pathogenic cascades upstream to Aβ-accumulation pathway. Thus, miR-9 and miR-181c target 3'-UTRs mRNA of a group of TGFBI, TRIM2, SIRT1, and BTBD3 proteins, important for brain homeostasis and disease pathogenesis [92].

### 2.2. MicroRNAs and Parkinson’s Disease

Typical pathological features of PD, the second most common neurodegenerative disorder, are loss of dopaminergic neurons in the *substantia nigra* and presence of Lewy bodies, intracellular inclusions that cause impaired neuron survival in several brain areas [93].

MiRNAs may act as regulators of both known and novel biological processes leading to PD (Table 1) [94,95,96].

Several studies showed the role of several microRNAs as modulator of the expression of α-synuclein. Due to the amplification of its gene locus, this protein is accumulated within the neurons, thus representing a major indicator of autosomal dominant PD. This emerges also in transgenic animal models where the over-expression of human α-synuclein caused impaired function or decreased survival of dopaminergic neurons [97].

miR-7 represses α-synuclein protein by targeting the 3'-UTR of α-synuclein mRNA [63,98,99]. Interestingly, miR-7 cooperates with miR-153 to control the quantity of α-synuclein produced, both in the adult brain and during neuronal development [63,98,99]. Alteration of α-synuclein expression is also associated to the increase of the translation of the fibroblast growth factor-20. This event is a consequence of the risk allele for rs12720208 polymorphism that disrupts the binding site for miR-433 [100,101].

miR-133b is specifically expressed in healthy midbrain dopaminergic neurons whereas is deficient in midbrain tissue from patients with PD. miR-133b targets transcription factor Pitx3 mRNA and regulates the maturation and function of midbrain dopaminergic neurons within a negative feedback circuit where Pitx3 is included (Figure 2). In fact, overexpression of a Pitx3 transgene lacking 3'-UTR regulatory element was able to “rescue” the reduced midbrain dopaminergic neurons. Moreover, consistent with this, the reduction of the transcription of the Dopamine Transporter by miR-133b was partially suppressed by over-expression of Pitx3 lacking the 3'-UTR miR-133b MRE sequences. Therefore, a model was proposed in which miR-133b plays a role in a feedback loop pathway, where Pitx3 specifically induces transcription of miR-133b and Pitx3 activity is downregulated by miR-133b at post-transcriptional level [64,102]. More recently, de Mena *et al.*, searching for DNA variants in miR-133 and PITX3 genes in PD patients (n = 777) and healthy controls (n = 650) from Spain, suggested that miR-133 and PITX3 gene variants did not contribute to the risk for PD, thus contrasting these data, at least in those specimens [67]. Additionally, miR-133b KO animal models were found to be devoid of dopaminergic neuron degeneration or impaired motor function, thereby indicating that a concert of miRNAs could be involved in a network that promote neuronal development and survival [103].

Down-regulation of miR-34b and miR-34c was associated to a decrease of Park-7 and Parkin, two proteins associated to familial and sporadic forms of PD (Figure 3) [65,66,104]. Additionally polymorphisms in the 3'-UTR of miR-34b and miR34c target the α-synuclein mRNA (Figure 3) [105].

Gain-of-function mutations in the leucine-rich repeat kinase 2 (LRRK2) mRNA cause age-dependent degeneration of dopaminergic neurons in both familial and sporadic PD. Interesting findings from LRRK2 studies in Drosophila demonstrated the interaction of LRRK2 with the RLC proteins dAgo1 or hAgo2. Within this network, in the brain of old fly, it was also observed that LRRK2 is involved in the negative regulation of dAgo1 levels and in the association of phospho-4E-BP1 with hAgo2 thus causing a deregulation of the synthesis of E2F1/DP and the impairment of miRNA pathway [66]. Of note was that the authors also demonstrated a concomitant role of let-7 or miR-184* in attenuating this aberrant circuit. Overall these findings delineate a trait of the pathogenic effect of LRRK2 [66].

### 2.3. MicroRNAs and Hungtinton’s Disease

Among the nine forms of Poly-glutamine (Poly-Q) disorders, HD is the most frequent. Here abnormal huntingtin (HTT) accumulation induces neuronal damage in brain patients. Increasing evidence revealed a key role of miRNAs in HD [70,74,106,107,108,109,110] (Table 1).

A widespread miRNAs dysregulation was mostly observed in the striatum and cortex [111], suggesting that miRNAs are involved in the impairment of transcriptome, which is a feature of HD [112]. In this regard, a pivotal role is exerted by RE1 Silencing Transcription Factor (REST), a direct and indirect regulator of a cohort of mRNAs and miRNAs altered in HD [68,112]. REST binds to several neuronal miRNA genes including miR-9/9*, miR-29b, miR-124, miR-132, miR-135, miR-137, and miR-153 [68,73]. Among the above miRNAs, miR-9 and miR-9* are direct target of REST and CoREST, respectively [69], whereas miR-222 is an indirect REST target [70].

Ghose *et al.* showed a down-regulation of miR-125b, miR-146a, and miR-150 in STHdh^Q111^/Hdh^Q111^ cells (HD mouse cell model) compared to the wild type counterpart. They demonstrated that miR-150 and miR-125b target p53 that decreases the NFκB (RelA/NFκB) p65 subunit expression and causes the reduction of miR-146a, thereby indicating that p53 directly or indirectly regulates the expression of miR-146a (Figure 2) [72]. Indeed, p53 is also regulated by miR-34b [113], which is significantly increased in the plasma of HD patients. *In vitro* studies proposed that the dysregulation of miR-34b could be a consequence of mHTT accumulation, at least in pluripotent and neurons differentiated human cells (Figure 3) [71].

Finally, miR-200a and miR-200c were found significantly upregulated at the pre-symptomatic stage of an HD mouse model. Comparative analysis of networks suggested that these two miRNAs may control genes involved in neuronal dysfunction induced by mutant HTT, such as atypical synaptic transmission and altered neurogenesis [74].

### 2.4. MicroRNAs and Amyotrophic Lateral Sclerosis

ALS is a neurodegenerative disease characterized by the loss of motor neurons, denervation of target muscles, muscle atrophy, and paralysis. Therefore, elucidating ALS pathogenesis may require the exploration of the bidirectional signaling between motor neurons and skeletal muscle fibers at neuromuscular synapses [51].

MiRNAs machinery has been found compromised in ALS (Table 1). For instance the absence of processed miRNAs, due to Dicer deletion in spinal motor neurons, resulted in a mouse model with progressive paralysis, astrocytosis, and signs of axonopathy, classical features of ALS [75]. Authors identified a single miR-9-binding site on the neurofilament light polypeptide (NFLP) mRNA and observed that miR-9 was also downregulated in Spinal Muscular Atrophy models thereby suggesting direct evidence for miRNAs malfunction in motor neuron diseases [75]. In another study, it was shown that the nuclear factor TDP-43, a major component of the inclusions in ALS patients and Frontotemporal Lobar Degenerative Disorder, was found associated with Drosha complex, thereby involving miRNAs biogenesis. In TDP-43^−/−^ mice let-7b was downregulated, and miR-663 upregulated [79]. This phenomenon was correlated with the expression of FUS/TLS suggesting that this complex acts as a cofactor involved in the biogenesis of a specific subset of miRNAs [114].

Of note, miRNAs dysregulation differ from the pre-symptomatic and the end-stage of ALS-SOD1 disease [115,116]. Analysis of the altered mitochondrial network genes in skeletal muscle revealed a contribution of miRNAs and that this kind of dysfunction plays a role in the progression of ALS. An accurate miRNAs expression study revealed a miR-23a, miR-29b, miR-206 and miR-455 upregulations in patients of ALS respect to control subjects [76]. miR-23a negatively regulates PGC-1α signaling, determining a significant reduction of PGC-1α, cytochome-b and COXIV protein levels in overexpressing miR-23a transgenic mice [76].

Recently, Morel *et al.* reported that the reduction of the expression of the glutamate transporter GLT1 in the end-stage SOD1 G93A mice, a mouse model of ALS, was a consequence of miR-124a activity. Interestingly they revealed an exosome-mediated transfer of microRNAs mechanism showing that miR-124a was transferred from neurons to astrocytes through neuronal exosomes [77]*.*

miR-206 is required for efficient regeneration of neuromuscular synapses after acute nerve injury, which probably accounts for its salutary effects in ALS [117]. Evidence indicated that, although mice genetically lacking miR-206 were able to engage normal neuromuscular synapses during development, deficiency of miR-206 in the ALS mouse model accelerates the disease progression, at least in part through the histone deacetylase 4 (HDAC4) and fibroblast growth factor signaling pathways (Figure 2) [78]. Remarkably, the phenotypes of miR-206 and HDAC4 mutant mice indicated that miR-206 and HDAC4 have opposite effects on retrograde signals required for the reinnervation. Additionally, fibroblast growth factor-7 (FGF7), FGF10, and FGF22, known to be regulators of synapse formation [118,119], were unaltered in miR-206^−/−^ mice compared to wild type animals, whereas the FGFBP1 protein (a factor that interacts with the FGF family members and enhance the FGF7 activity in rat L6 myoblasts by releasing the FGF up-taken by the extracellular matrix [120]) was downregulated in muscles of miR-206^−/−^ mice and upregulated in muscles of HDAC4^−/−^ mice after denervation. Collectively, these findings suggest that miR-206 and HDAC4 help and block the innervations of neuromuscular junction, respectively, via opposing effects on FGFBP1 [78].

### 2.5. MicroRNAs and Lysosomal Storage Disorders

LSDs are a group of inherited lysosomal metabolic disorders caused by deficiencies of lysosomal enzymes and of a series of lysosomal proteins, which are involved in the abnormal accumulation of undegraded substrates. These disorders are characterized by a broad spectrum of clinical variants and of progressive lethal neurodegeneration [53,121,122,123,124].

Recent studies point to miRNAs as tool for gene therapy application [117]. In this regard we have developed the hematopoietic stem cell (HSC)-based gene therapy for Krabbe disease, fatal LSD caused by mutations in the galactocerebrosidase (GALC) gene [125,126]. To be effective, this therapy needs the modulation of the transgene expression to avoid forced GALC toxicity [127]. The rationale of this approach was the inhibition of GALC toxicity in the HSCs used for GLD treatment in order to protect differentiated cells that could be able to carry efficiently their function. To this end, we took advantage of this by the observation that miR-126 and miR-130 were expressed in HSCs but not in differentiated progeny. By incorporating miR-126 target sequences into a GALC-expressing lentiviral vector we have suppressed GALC expression in HSCs maintaining a robust expression in mature hematopoietic cells [80].

In another approach, Osborn *et al.* have used the same strategy to achieve an opposite goal. To develop a Mucopolysaccharidosis type I (MPS I) treatment, a LSD characterized by a progressive accumulation of glycosaminoglycans due to the α-L-iduronidase (IDUA) gene mutation, they have included a miRNA sequence in a minicircle DNA vector with a tissue-specific promoter to obtain a slight improvement of its long-term expression respect to the same vector without the miRNA element. This therapy allowed the restoration of IDUA enzyme and the amelioration of pathology in MPS I mice although with the support of immune modulation by costimulatory blockade [128].

A late endosomal-lysosomal accumulation of multiple lipid molecules characterizes the Niemann-Pick type C (NPC) disease. MiRNAs pattern, both in health subjects than in NPC patients, have been analyzed in human fibroblasts by Ozsait *et al.* using a TaqMan Low-Density Array system containing 365 mature human miRNAs. They showed that NPC patient fibroblasts presented altered levels of miRNAs pattern respect to controls. Remarkably, they found that miR-196a, miR-196b, and miR-296 were upregulated whereas 38 miRNAs were significantly downregulated in NPC cells. Among these non-coding RNAs, miR-98 and miR-143, the lipid biosynthesis associated miRNA, were the most downregulated [81]. 

## 3. Concluding Remarks

The implications of miRNAs in neurodegenerative molecular mechanisms offer the opportunity to elucidate the pathogenesis of the nervous system diseases.

As described in this review, several miRNAs could be involved in the same molecular network. For instance, miR-150, miR-125b [72], and miR-34b [71], although with different mechanisms, altered the p53 homeostasis in HD. Additionally, miR-34b and miR-34c down-regulation was associated to PD [65,105], whereas miR-34c [87] and miR-34a [89] correlated with AD. Remarkably, the work by Liu and collaborators associated the activity of miR-34 on the control of age-associated events and long-term brain integrity in *Drosophila*, and suggested that miR-34 could be a molecular link between ageing and neurodegeneration [129].

Alternatively, abnormal expression of a single miRNA molecule could be responsible of a specific pathology. Thus, miR-206 seems to be implicated in ALS [78] while miR-133b could be involved in PD pathogenesis [64,102].

Of note, specific antisense antagomirs (miRNA inhibitors) could be used to affect the activity of specific miRNAs and to develop a therapeutic strategy for these disorders. 

Therefore, miRNAs-based therapeutics has greatly advanced because of their capability to efficiently silence multiple messages concurrently within an entire disease pathway.
